# *N*,*N*′-Bis(pyridin-2-yl)benzene-1,4-diamine–quinoxaline (2/1)

**DOI:** 10.1107/S1600536811046356

**Published:** 2011-11-09

**Authors:** Barbara Wicher, Maria Gdaniec

**Affiliations:** a Faculty of Chemistry, Adam Mickiewicz University, 60-780 Poznań, Poland

## Abstract

The asymmetric unit of the title compound, 2C_16_H_14_N_4_·C_8_H_6_N_2_, consits of one mol­ecule of *N*,*N*′-bis­(pyridin-2-yl)benzene-1,4-diamine (PDAB) and one half-mol­ecule of quinoxaline (QX) that is located around an inversion centre and disordered over two overlapping positions. The PDAB mol­ecule adopts a non-planar conformation with an *E* configuration at the two partially double *exo* C N bonds of the 2-pyridyl­amine units. In the crystal, these self-complementary units are N—H⋯N hydrogen bonded *via* a cyclic *R*_2_^2^(8) motif, creating tapes of PDAB mol­ecules extending along [010]. Inversion-related tapes are arranged into pairs through π–π stacking inter­actions between the benzene rings [centroid–centroid distance = 3.818 (1) Å] and the two symmetry-independent pyridine groups [centroid–centroid distance = 3.760 (1) Å]. The QX mol­ecules are enclosed in a cavity formed between six PDAB tapes.

## Related literature

For the structures of polymorphic forms of *N*,*N*′-di(pyridin-2-yl)benzene-1,4-diamine and its co-crystal with phenazine, see: Bensemann *et al.* (2002 ▶); Wicher & Gdaniec (2011 ▶); Gdaniec *et al.* (2005 ▶).
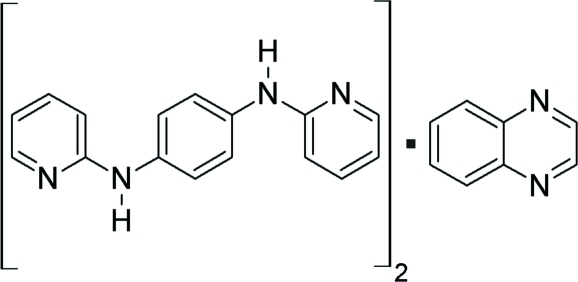


## Experimental

### 

#### Crystal data

2C_16_H_14_N_4_·C_8_H_6_N_2_
*M* *_r_* = 654.77
Monoclinic, 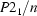

*a* = 11.8285 (9) Å
*b* = 9.1223 (7) Å
*c* = 14.7952 (9) Å
β = 93.698 (5)°
*V* = 1593.1 (2) Å^3^
*Z* = 2
Mo *K*α radiation
μ = 0.09 mm^−1^
*T* = 130 K
0.50 × 0.30 × 0.25 mm

#### Data collection

Kuma KM-4-CCD κ-geometry diffractometer
8116 measured reflections
2897 independent reflections
2082 reflections with *I* > 2σ(*I*)
*R*_int_ = 0.033

#### Refinement

*R*[*F* ^2^ > 2σ(*F* ^2^)] = 0.045
*wR*(*F* ^2^) = 0.121
*S* = 0.97
2897 reflections
226 parameters
H-atom parameters constrained
Δρ_max_ = 0.22 e Å^−3^
Δρ_min_ = −0.28 e Å^−3^

### 

Data collection: *CrysAlis CCD* (Oxford Diffraction, 2002 ▶); cell refinement: *CrysAlis RED* (Oxford Diffraction, 2002 ▶); data reduction: *CrysAlis RED*; program(s) used to solve structure: *SHELXS97* (Sheldrick, 2008 ▶); program(s) used to refine structure: *SHELXL97* (Sheldrick, 2008 ▶); molecular graphics: *ORTEP-3 for Windows* (Farrugia, 1997 ▶) and *Mercury* (Macrae *et al.*, 2006 ▶); software used to prepare material for publication: *SHELXL97*.

## Supplementary Material

Crystal structure: contains datablock(s) global, I. DOI: 10.1107/S1600536811046356/rz2662sup1.cif

Structure factors: contains datablock(s) I. DOI: 10.1107/S1600536811046356/rz2662Isup2.hkl

Additional supplementary materials:  crystallographic information; 3D view; checkCIF report

## Figures and Tables

**Table 1:** Hydrogen-bond geometry (Å, °)

*D*—H⋯*A*	*D*—H	H⋯*A*	*D*⋯*A*	*D*—H⋯*A*
N14—H14*N*⋯N2^i^	0.90	2.12	2.9998 (17)	166
N7—H7*N*⋯N16^ii^	0.90	2.13	3.0173 (18)	167

Symmetry codes: (i) 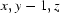; (ii) 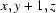.

**Fig. 1.**
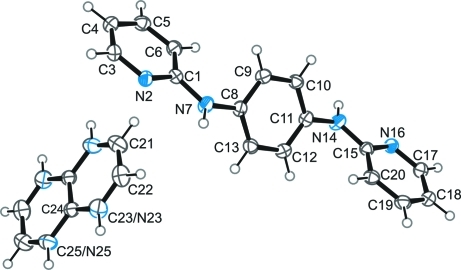
: Asymmetric unit of the title compound with displacement ellipsoids shown at the 50% probability level. The elipsoids representing positions occupied equally by C and N atoms are coloured in grey and blue. The unlabelled atoms of quinoxaline are related to the labelled one by the symmetry operation: 1 - x,1 - y,1 - z.

**Fig. 2.**
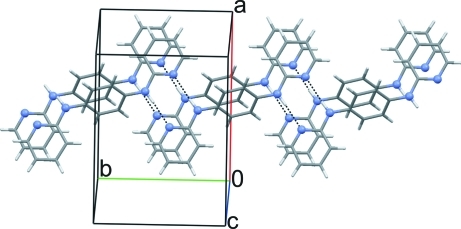
: π-π stacking interactions connecting hydrogen-bonded PDAB tapes into pairs. N—H···N hydrogen bonds are shown as dashed lines.

**Fig. 3.**
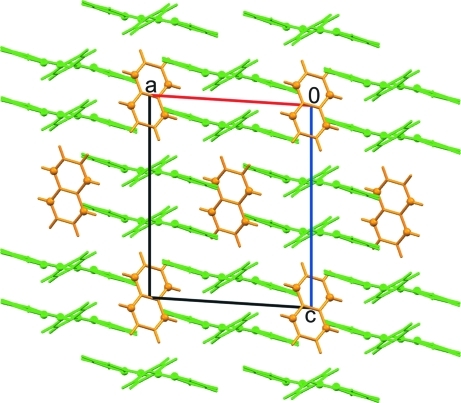
: Crystal packing diagram viewed along b illustrating arrangement of the hydrogen-bonded tapes of PDAB in the crystal and quinoxaline molecules enclosed in the cavity formed between the tapes.

## supplementary crystallographic information

### Comment

*N*,*N*'-Di(pyridin-2-yl)benzene-1,4-diamine (PDAB) is a very versatile supramolecular reagent. It has been shown that it can cocrystallize with the aromatic base, phenazine, forming cocrystals with the 1:4 molar ratio (Gdaniec *et al.*, 2005). In this cocrystal the PDAB molecule is centrosymmetric and adopts a nearly planar conformation and a *Z,Z* form, *i.e.* the configuration at the partially double *exo* C≐ N bonds of its two 2-pyridylamine units is *Z*. The PDAB molecules are hydrogen bonded to phenazine molecules but, most importantly, their π-faces are directed to the edges of the phenazine molecules arranged *via*π-π stacking interactions into quartets. To check whether a similar packing motif will be observed for a compound containing the pyrazine fragment but a reduced π-system compared to phenazine, an attempt was made to cocrystallize PDAB with quinoxaline (QX). Cocrystallization was successful when PDAB was dissolved in molten QX (m.p. 301 K) and the solution was slowly evaporated at 331 K yielding the title molecular complex with 2:1 PDAB/QX ratio (Fig. 1). In contrast with the PDAB/phenazine cocrystal, in the title complex the PDAB molecule is nonplanar and adopts an *E*,*E* form that promotes formation of a cyclic *R*^2^_2_(8) motif *via* N—H···N hydrogen bond between the self-complementary 2-pyridylamine units (Table 1). These cyclic motifs assemble PDAB molecules into tapes extending along [010]. The tapes related by inversion center are arranged into pairs through π-π stacking interactions between the benzene rings [centroid-centroid distance 3.818 (1) Å] and the two symmetry independent pyridine groups [centroid-centroid distance 3.760 (1) Å] (Fig. 2). Similar tape motifs have been observed in two of the three PDAB polymorphs (Bensemann *et al.*, 2002; Wicher & Gdaniec, 2011), however these polymorphic structures were not stabilized by π-π stacking interactions between the tapes.

The QX molecule, that is not hydrogen bonded to PDAB, is enclosed in a centrosymmetric cavity formed between six PDAB tapes (Fig. 3). This leads to a disorder of the non-centrosymmetric QX molecule which in the cavity is located, with equal occupancies, in two alternative overlapping positions. Thus QX molecule in this crystal structure simulates the shape of a naphthalene molecule.

As there are no specific interactions between QX and PDAB molecules the driving force for the complex formation with PDAB is different in the two cocrystals with the aromatic heterobases containing the pyrazine ring.

### Experimental

*N*,*N*'-Di(pyridin-2-yl)benzene-1,4-diamine (0.07 g, 0.27 mmol) was dissolved in an excess of the melted quinoxaline. The solution was heated at 331 K and after a few days colourless crystal suitable for X-ray analysis were obtained.

### Refinement

All H atoms were located in difference electron-density maps, however for further refinement their positions were determined geometrically with N—H and C—H bond lengths of 0.90 Å and 0.95 Å, respectively. All H atoms were refined in the riding-model approximation, with *U*_iso_(H)=1.2*U*_eq_(N,*C*).

### Crystal data

**Table:**

2C_16_H_14_N_4_·C_8_H_6_N_2_	*F*(000) = 688
*M**_r_* = 654.77	*D*_x_ = 1.365 Mg m^−^^3^
Monoclinic, *P*2_1_/*n*	Mo *K*α radiation, λ = 0.71073 Å
Hall symbol: -P 2yn	Cell parameters from 2533 reflections
*a* = 11.8285 (9) Å	θ = 4.1–25.0°
*b* = 9.1223 (7) Å	µ = 0.09 mm^−^^1^
*c* = 14.7952 (9) Å	*T* = 130 K
β = 93.698 (5)°	Prism, colourless
*V* = 1593.1 (2) Å^3^	0.50 × 0.30 × 0.25 mm
*Z* = 2	

### Data collection

**Table:**

Kuma KM-4-CCD κ-geometry diffractometer	2082 reflections with *I* > 2σ(*I*)
Radiation source: fine-focus sealed tube	*R*_int_ = 0.033
graphite	θ_max_ = 25.4°, θ_min_ = 4.1°
ω scans	*h* = −13→14
8116 measured reflections	*k* = −10→9
2897 independent reflections	*l* = −17→17

### Refinement

**Table:**

Refinement on *F*^2^	Primary atom site location: structure-invariant direct methods
Least-squares matrix: full	Secondary atom site location: difference Fourier map
*R*[*F*^2^ > 2σ(*F*^2^)] = 0.045	Hydrogen site location: inferred from neighbouring sites
*wR*(*F*^2^) = 0.121	H-atom parameters constrained
*S* = 0.97	*w* = 1/[σ^2^(*F*_o_^2^) + (0.0796*P*)^2^] where *P* = (*F*_o_^2^ + 2*F*_c_^2^)/3
2897 reflections	(Δ/σ)_max_ < 0.001
226 parameters	Δρ_max_ = 0.22 e Å^−^^3^
0 restraints	Δρ_min_ = −0.28 e Å^−^^3^

### Special details

**Table:**

Geometry. All e.s.d.'s (except the e.s.d. in the dihedral angle between two l.s. planes) are estimated using the full covariance matrix. The cell e.s.d.'s are taken into account individually in the estimation of e.s.d.'s in distances, angles and torsion angles; correlations between e.s.d.'s in cell parameters are only used when they are defined by crystal symmetry. An approximate (isotropic) treatment of cell e.s.d.'s is used for estimating e.s.d.'s involving l.s. planes.
Refinement. Refinement of *F*^2^ against ALL reflections. The weighted *R*-factor *wR* and goodness of fit *S* are based on *F*^2^, conventional *R*-factors *R* are based on *F*, with *F* set to zero for negative *F*^2^. The threshold expression of *F*^2^ > σ(*F*^2^) is used only for calculating *R*-factors(gt) *etc*. and is not relevant to the choice of reflections for refinement. *R*-factors based on *F*^2^ are statistically about twice as large as those based on *F*, and *R*- factors based on ALL data will be even larger.

### Fractional atomic coordinates and isotropic or equivalent isotropic displacement parameters (Å^2^)

**Table:**

	*x*	*y*	*z*	*U*_iso_*/*U*_eq_	Occ. (<1)
N2	0.34420 (10)	0.46004 (13)	0.12792 (9)	0.0251 (3)	
N7	0.44563 (11)	0.24793 (13)	0.12278 (9)	0.0291 (3)	
H7N	0.5037	0.3082	0.1126	0.035*	
N14	0.55190 (10)	−0.35168 (13)	0.12548 (9)	0.0295 (3)	
H14N	0.4941	−0.4122	0.1359	0.035*	
N16	0.65420 (10)	−0.56243 (13)	0.11839 (9)	0.0260 (3)	
C1	0.34338 (12)	0.31287 (16)	0.13482 (10)	0.0232 (4)	
C3	0.24879 (13)	0.53106 (17)	0.14294 (10)	0.0282 (4)	
H3	0.2492	0.6349	0.1382	0.034*	
C4	0.15003 (13)	0.46477 (18)	0.16475 (11)	0.0301 (4)	
H4	0.0841	0.5205	0.1745	0.036*	
C5	0.15028 (13)	0.31317 (18)	0.17200 (11)	0.0292 (4)	
H5	0.0839	0.2630	0.1876	0.035*	
C6	0.24654 (13)	0.23600 (17)	0.15651 (10)	0.0266 (4)	
H6	0.2474	0.1321	0.1604	0.032*	
C8	0.46942 (13)	0.09666 (16)	0.12475 (10)	0.0243 (4)	
C9	0.40085 (12)	−0.00484 (17)	0.07789 (10)	0.0264 (4)	
H9	0.3335	0.0269	0.0452	0.032*	
C10	0.42953 (13)	−0.15119 (16)	0.07830 (10)	0.0256 (4)	
H10	0.3813	−0.2195	0.0463	0.031*	
C11	0.52812 (13)	−0.20032 (16)	0.12488 (10)	0.0253 (4)	
C12	0.59638 (13)	−0.09904 (17)	0.17195 (11)	0.0272 (4)	
H12	0.6637	−0.1307	0.2047	0.033*	
C13	0.56739 (13)	0.04714 (17)	0.17172 (10)	0.0270 (4)	
H13	0.6152	0.1152	0.2042	0.032*	
C15	0.65386 (13)	−0.41581 (16)	0.11138 (10)	0.0239 (4)	
C17	0.75049 (13)	−0.63284 (18)	0.10303 (11)	0.0302 (4)	
H17	0.7507	−0.7367	0.1074	0.036*	
C18	0.84841 (14)	−0.56538 (18)	0.08156 (11)	0.0310 (4)	
H18	0.9149	−0.6201	0.0719	0.037*	
C19	0.84671 (13)	−0.41393 (18)	0.07442 (11)	0.0303 (4)	
H19	0.9129	−0.3628	0.0594	0.036*	
C20	0.75006 (13)	−0.33812 (17)	0.08900 (10)	0.0276 (4)	
H20	0.7482	−0.2343	0.0840	0.033*	
C21	0.42626 (15)	0.38226 (19)	0.36086 (12)	0.0393 (5)	
H21	0.3839	0.3310	0.3142	0.047*	
C22	0.53039 (16)	0.44592 (18)	0.34188 (13)	0.0410 (5)	
H22	0.5570	0.4363	0.2829	0.049*	
N23	0.59282 (13)	0.51949 (17)	0.40527 (11)	0.0368 (4)	0.50
C23	0.59282 (13)	0.51949 (17)	0.40527 (11)	0.0368 (4)	0.50
H23	0.6629	0.5629	0.3922	0.044*	0.50
C24	0.55231 (12)	0.53158 (16)	0.49075 (11)	0.0275 (4)	
C25	0.61465 (13)	0.60804 (16)	0.55764 (11)	0.0357 (4)	0.50
H25	0.6846	0.6529	0.5457	0.043*	0.50
N25	0.61465 (13)	0.60804 (16)	0.55764 (11)	0.0357 (4)	0.50

### Atomic displacement parameters (Å^2^)

**Table:**

	*U*^11^	*U*^22^	*U*^33^	*U*^12^	*U*^13^	*U*^23^
N2	0.0240 (7)	0.0225 (7)	0.0290 (7)	0.0005 (5)	0.0025 (6)	−0.0026 (5)
N7	0.0227 (7)	0.0202 (7)	0.0449 (9)	−0.0022 (5)	0.0067 (6)	−0.0004 (6)
N14	0.0224 (7)	0.0208 (7)	0.0457 (9)	−0.0006 (5)	0.0056 (6)	0.0047 (6)
N16	0.0248 (7)	0.0229 (7)	0.0301 (8)	0.0004 (5)	0.0011 (6)	−0.0004 (6)
C1	0.0249 (8)	0.0223 (8)	0.0223 (8)	−0.0012 (6)	0.0010 (6)	−0.0020 (6)
C3	0.0301 (9)	0.0246 (8)	0.0298 (9)	0.0026 (7)	0.0012 (7)	−0.0040 (7)
C4	0.0243 (8)	0.0339 (9)	0.0323 (9)	0.0032 (7)	0.0030 (7)	−0.0048 (7)
C5	0.0247 (8)	0.0361 (9)	0.0270 (9)	−0.0044 (7)	0.0026 (7)	−0.0009 (7)
C6	0.0283 (9)	0.0239 (8)	0.0277 (9)	−0.0022 (7)	0.0019 (7)	0.0000 (6)
C8	0.0238 (8)	0.0212 (8)	0.0284 (9)	−0.0007 (6)	0.0059 (7)	0.0001 (6)
C9	0.0241 (8)	0.0258 (8)	0.0292 (9)	0.0000 (7)	0.0004 (7)	0.0024 (7)
C10	0.0244 (8)	0.0253 (8)	0.0271 (9)	−0.0034 (6)	0.0010 (6)	−0.0015 (6)
C11	0.0257 (8)	0.0219 (8)	0.0289 (9)	0.0009 (6)	0.0056 (7)	0.0024 (6)
C12	0.0231 (8)	0.0299 (9)	0.0283 (9)	0.0019 (7)	0.0003 (7)	0.0026 (7)
C13	0.0248 (8)	0.0277 (9)	0.0288 (9)	−0.0034 (7)	0.0034 (7)	−0.0035 (7)
C15	0.0242 (8)	0.0246 (8)	0.0225 (8)	0.0002 (6)	−0.0007 (6)	0.0002 (6)
C17	0.0298 (9)	0.0267 (8)	0.0336 (9)	0.0037 (7)	−0.0010 (7)	−0.0037 (7)
C18	0.0259 (9)	0.0337 (9)	0.0334 (9)	0.0028 (7)	0.0011 (7)	−0.0068 (7)
C19	0.0231 (9)	0.0374 (10)	0.0302 (9)	−0.0054 (7)	0.0012 (7)	−0.0031 (7)
C20	0.0273 (9)	0.0259 (8)	0.0292 (9)	−0.0028 (7)	−0.0004 (7)	0.0008 (7)
C21	0.0462 (11)	0.0331 (10)	0.0372 (11)	−0.0056 (8)	−0.0086 (9)	0.0026 (8)
C22	0.0550 (12)	0.0324 (10)	0.0362 (10)	0.0004 (9)	0.0081 (9)	0.0027 (8)
N23	0.0364 (9)	0.0337 (9)	0.0411 (10)	−0.0045 (7)	0.0092 (7)	0.0025 (7)
C23	0.0364 (9)	0.0337 (9)	0.0411 (10)	−0.0045 (7)	0.0092 (7)	0.0025 (7)
C24	0.0263 (8)	0.0214 (8)	0.0344 (9)	0.0003 (6)	−0.0005 (7)	0.0021 (7)
C25	0.0314 (8)	0.0331 (9)	0.0417 (10)	−0.0057 (7)	−0.0055 (7)	0.0031 (7)
N25	0.0314 (8)	0.0331 (9)	0.0417 (10)	−0.0057 (7)	−0.0055 (7)	0.0031 (7)

### Geometric parameters (Å, °)

**Table:**

N2—C3	1.3324 (18)	C11—C12	1.385 (2)
N2—C1	1.3465 (19)	C12—C13	1.377 (2)
N7—C1	1.3686 (19)	C12—H12	0.9500
N7—C8	1.4083 (19)	C13—H13	0.9500
N7—H7N	0.9001	C15—C20	1.398 (2)
N14—C15	1.3685 (19)	C17—C18	1.367 (2)
N14—C11	1.4090 (19)	C17—H17	0.9500
N14—H14N	0.9000	C18—C19	1.386 (2)
N16—C17	1.3397 (19)	C18—H18	0.9500
N16—C15	1.3415 (19)	C19—C20	1.365 (2)
C1—C6	1.398 (2)	C19—H19	0.9500
C3—C4	1.372 (2)	C20—H20	0.9500
C3—H3	0.9500	C21—N25^i^	1.331 (2)
C4—C5	1.387 (2)	C21—C25^i^	1.331 (2)
C4—H4	0.9500	C21—C22	1.406 (3)
C5—C6	1.370 (2)	C21—H21	0.9499
C5—H5	0.9500	C22—N23	1.336 (2)
C6—H6	0.9500	C22—H22	0.9500
C8—C9	1.387 (2)	N23—C24	1.385 (2)
C8—C13	1.388 (2)	N23—H23	0.9500
C9—C10	1.377 (2)	C24—C25	1.384 (2)
C9—H9	0.9500	C24—C24^i^	1.408 (3)
C10—C11	1.390 (2)	C25—C21^i^	1.331 (2)
C10—H10	0.9500	C25—H25	0.9499
			
C3—N2—C1	117.51 (13)	C11—C12—H12	119.7
C1—N7—C8	126.76 (13)	C12—C13—C8	121.08 (14)
C1—N7—H7N	116.6	C12—C13—H13	119.5
C8—N7—H7N	116.6	C8—C13—H13	119.5
C15—N14—C11	126.54 (13)	N16—C15—N14	114.37 (13)
C15—N14—H14N	116.8	N16—C15—C20	121.68 (14)
C11—N14—H14N	116.7	N14—C15—C20	123.92 (14)
C17—N16—C15	117.59 (14)	N16—C17—C18	124.47 (15)
N2—C1—N7	114.27 (13)	N16—C17—H17	117.8
N2—C1—C6	121.83 (14)	C18—C17—H17	117.8
N7—C1—C6	123.84 (14)	C17—C18—C19	117.27 (15)
N2—C3—C4	124.61 (15)	C17—C18—H18	121.4
N2—C3—H3	117.7	C19—C18—H18	121.4
C4—C3—H3	117.7	C20—C19—C18	120.08 (15)
C3—C4—C5	117.38 (15)	C20—C19—H19	120.0
C3—C4—H4	121.3	C18—C19—H19	120.0
C5—C4—H4	121.3	C19—C20—C15	118.89 (15)
C6—C5—C4	119.79 (15)	C19—C20—H20	120.6
C6—C5—H5	120.1	C15—C20—H20	120.6
C4—C5—H5	120.1	N25^i^—C21—C22	121.85 (16)
C5—C6—C1	118.86 (15)	C25^i^—C21—C22	121.85 (16)
C5—C6—H6	120.6	N25^i^—C21—H21	119.1
C1—C6—H6	120.6	C25^i^—C21—H21	119.1
C9—C8—C13	118.40 (14)	C22—C21—H21	119.1
C9—C8—N7	122.29 (14)	N23—C22—C21	121.25 (17)
C13—C8—N7	119.25 (13)	N23—C22—H22	119.3
C10—C9—C8	120.60 (14)	C21—C22—H22	119.4
C10—C9—H9	119.7	C22—N23—C24	118.20 (15)
C8—C9—H9	119.7	C22—N23—H23	121.1
C9—C10—C11	120.88 (14)	C24—N23—H23	120.7
C9—C10—H10	119.6	C25—C24—N23	119.55 (14)
C11—C10—H10	119.6	C25—C24—C24^i^	120.10 (19)
C12—C11—C10	118.52 (14)	N23—C24—C24^i^	120.34 (18)
C12—C11—N14	122.74 (14)	C21^i^—C25—C24	118.25 (15)
C10—C11—N14	118.68 (13)	C21^i^—C25—H25	120.8
C13—C12—C11	120.52 (14)	C24—C25—H25	120.9
C13—C12—H12	119.7		
			
C3—N2—C1—N7	−177.03 (13)	C11—C12—C13—C8	0.1 (2)
C3—N2—C1—C6	0.3 (2)	C9—C8—C13—C12	0.1 (2)
C8—N7—C1—N2	−178.90 (14)	N7—C8—C13—C12	−177.06 (14)
C8—N7—C1—C6	3.9 (2)	C17—N16—C15—N14	178.27 (13)
C1—N2—C3—C4	−0.1 (2)	C17—N16—C15—C20	−0.1 (2)
N2—C3—C4—C5	0.3 (2)	C11—N14—C15—N16	178.07 (14)
C3—C4—C5—C6	−0.7 (2)	C11—N14—C15—C20	−3.6 (2)
C4—C5—C6—C1	0.9 (2)	C15—N16—C17—C18	0.6 (2)
N2—C1—C6—C5	−0.7 (2)	N16—C17—C18—C19	−0.7 (2)
N7—C1—C6—C5	176.37 (14)	C17—C18—C19—C20	0.3 (2)
C1—N7—C8—C9	47.3 (2)	C18—C19—C20—C15	0.2 (2)
C1—N7—C8—C13	−135.63 (16)	N16—C15—C20—C19	−0.3 (2)
C13—C8—C9—C10	0.1 (2)	N14—C15—C20—C19	−178.51 (14)
N7—C8—C9—C10	177.18 (14)	N25^i^—C21—C22—N23	−0.1 (3)
C8—C9—C10—C11	−0.6 (2)	C25^i^—C21—C22—N23	−0.1 (3)
C9—C10—C11—C12	0.8 (2)	C21—C22—N23—C24	0.0 (3)
C9—C10—C11—N14	178.09 (14)	C22—N23—C24—C25	−179.52 (15)
C15—N14—C11—C12	−48.1 (2)	C22—N23—C24—C24^i^	−0.4 (3)
C15—N14—C11—C10	134.72 (16)	N23—C24—C25—C21^i^	−179.92 (15)
C10—C11—C12—C13	−0.6 (2)	C24^i^—C24—C25—C21^i^	1.0 (3)
N14—C11—C12—C13	−177.76 (14)		

Symmetry codes: (i) −*x*+1, −*y*+1, −*z*+1.

### Hydrogen-bond geometry (Å, °)

**Table:**

*D*—H···*A*	*D*—H	H···*A*	*D*···*A*	*D*—H···*A*
N14—H14N···N2^ii^	0.90	2.12	2.9998 (17)	166
N7—H7N···N16^iii^	0.90	2.13	3.0173 (18)	167

Symmetry codes: (ii) *x*, *y*−1, *z*; (iii) *x*, *y*+1, *z*.
